# Case cancellations and associated factors on the day of surgery in hospitals of Wolaita Zone, South Ethiopia

**DOI:** 10.1186/s12893-024-02330-5

**Published:** 2024-02-04

**Authors:** Tadele Lankrew Ayalew

**Affiliations:** Department of Nursing, College of medicine and health science, Wolaita Sodo University, Wolaita, Ethiopia

**Keywords:** Cancellation, Cases, Elective surgery, Prevalence, Ethiopia

## Abstract

**Background:**

Cancellations of elective surgery cases are frequent and have significant negative consequences. It causes wasting of valuable resources, patient unhappiness, and psychological stress of patients. Despite this, little is known about the case cancellation and associated factors on the day of surgery in Ethiopia, particularly in the study area.

**Objective:**

This study aimed to assess the magnitude of case cancellation and associated factors on the day of surgery in hospitals in Wolaita zone, South Ethiopia, from May 17 to June 17, 2023.

**Methods:**

A hospital-based cross-sectional study involving 322 patients was conducted at Wolaita Sodo Zone, South Ethiopia. All elective surgical cases scheduled during the study period were included. The entire number of participants was selected using a systematic random sampling process. Epidata V.3 was used to enter data, and SPSS V.25 was used to analyze it. Binary logistic regression was used to check for a possible association. *P*-values < 0.05 and 95% CI were used on multi-variable analysis as the threshold for the significant statistical association.

**Result:**

A total of 313 study participants were scheduled for elective surgical procedures during the study period and gave a response rate of 97.2%. The mean (± SD) age of the study participants was 39.18 (± 10.64) years. The two-third of patients, 53(64%) were rural residents, and more than half (178, or 55.3%) of the participants were female. This finding showed that the case cancellation was 22.4% (95% CI: 19.3 -25.9%). Among the total canceled cases, 49(58.3%) were males. Variables like rural residence (AOR = 3.48 95% CI: 1.22–9.95), Lack of lab result (AOR = 2.33, 95%CI:1.20–4.51), ophthalmology dept. (AOR = 2.53 95% CI:1.52–4.49), HTN (AOR = 2.53, 95% CI:1.52–4.49), patient refusal (AOR = 3.01 95% CI:1.22–5.05), and age b/n 31 and 43 (AOR = 1.50, 95% CI:1.02–2.01) were significantly associated factors with cancellation of elective surgical cases.

**Conclusion:**

In this study schedule of case cancellation was high. The contributing factors of case cancellation were rural residence, Lack of lab results, ophthalmology dept, HTN, patient refusal, and age. To decrease unnecessary cancellations and increase cost efficiency, hospital administration and medical staff must plan ahead carefully, communicate effectively, and make efficient use of hospital resources.

## Introduction

Elective surgery cancellations were defined as elective surgeries that were scheduled on the final surgeries list for the day or those later added to the list but not performed that day were considered cancellations of elective procedures [1, 2]. A planned surgery cancellation will occur if the procedure that was planned for that day on the Last Surgery List or finally on the list is not done on that day [2]. The following categories were used to group the reasons for stopping: Potentially avoidable (no surgery time, no post-operative bed, listing errors, administrative cases, equipment or transport problems, communication errors, patient not ready, surgeons absent) or unavoidable (patient cancellation, patient clinical change, emergency priority, patient unprepared, surgeon absent) [3, 4].

Elective surgery cancellations are a significant problem worldwide due to lack of operating room time, administrative issues, and lack of operating rooms and equipment. Canceling elective surgeries is an inefficient use of surgical time and a waste of resources [5–7]. In developing countries with limited resources due to poor socioeconomic conditions, most hospitals face frequent cancellations of scheduled surgeries [8].

Previous studies in Ethiopia have shown dropout rates due to elective surgery to range from 15.23% in Gondar Hospital to 33.9% in Black Lion Hospital [6, 8] respectively. On the other hand the magnitude of surgical cancelation in Wolaita Sodo referral hospital 25.6% [6], in public hospitals of Harar region 35.2% [9], in Debretabor general hospital 32.1% [10], in Iran 6.3% [11], and a systematic review and meta-analysis done in Ethiopia was 21.41% [12].

Canceling a planned surgical procedure causes significant psychological trauma to the patient, family, and community. Various studies have shown that withdrawing patients from elective surgery increases costs, reduces efficiency, doubles the workload, and wastes time in the operating room. Cancellations of planned surgeries always lead to underutilization of human and hospital resources, increasing the cost of patient care through longer hospital stays [2, 13, 14].

Repeated cancellations have been found to negatively impact patient satisfaction, staff morale, staff-patient relationships, operating room resources, and perceived quality of care [1]. In general, case cancellations due to long hospital stays impact on resources and increase medical costs [15].

Cancellation of elective surgery is a major problem with many adverse consequences [6, 16]. Cancellations of elective surgery led to a waste of health care resources, increases costs of operating rooms, resulted in wasted operating room time and decreases efficacy, decrease patient satisfaction, and undermine the morale of staff [9, 17].

There is substantial evidence that cancellation of elective surgery has significant psychosocial and economic impacts on patients and their families. Besides, it affects the health care delivery and revenue of the hospital, which entails mitigating strategies to prevent avoidable surgical cancellations [18, 19]. Identification of reasons for elective surgical case cancelation can be able the management body to make appropriate strategies and make better use of its operation theater facility [20, 21]. So, conducting this research may increase the awareness, the sensitivity of the problem to health professionals, and hospital management for better management of the problem at any level. In addition to this, the result of the study might be advantageous to motivate and simulate more detailed research. Studies on the magnitude and reason of case cancellation in Ethiopia are limited; especially since the study is not conducted in our setup. Therefore, this study aimed to assess the magnitude and associated factors of case cancellation at Wolaita Sodo Zone hospitals, in south Ethiopia.

## Methods and material

### Study area

The study was conducted at Wolaita Sodo Zonal Hospitals which is found in South Ethiopia which is located at about 328-kilometers from Addis Ababa and 154KM from regional City Hawassa. The hospital gives service of elective surgery in different departments those includes’; General surgery, orthopedic surgery, urologic surgery, obstetrics and gynecologic surgery and maxillofacial surgery. Elective surgeries were performed from Monday to Friday.

### Study design and period

A hospital-based cross-sectional study was conducted from May 17 to June 17, 2023 to assess the magnitude and associated factors of surgical case cancellation.

### Source population

All surgical cases that undergo surgery in Wolaita Zonal hospitals.

### Study populations

All patients scheduled for different elective surgical procedures in selected Wolaita Zonal hospitals during the study period.

### Inclusion and exclusion criteria

All patients scheduled for different elective surgery with full information containing age, sex, planned procedure and date of the surgery with their diagnosis were included in the study.

Individuals who were listed for elective surgery but were done before the day of schedule as emergency and patients scheduled in a minor operating room for minor surgery were excluded from the study.

### Sampling and sampling size

The sample size was determined by using a single population proportion formula by considering the following assumptions: *p*-value of 25.6% (0.256) [2] for case cancellation done by Wolaita Sodo Comprehensive Specialized hospital, 95% (1.96) of confidence interval and 5% (0.05) margin of error ($n=\frac{\left({\frac{z\alpha }{2})}^{2 }p*q\right)}{{d}^{2}}$). The sample size for this study was obtained by adding a 10% non-response rate, and the final sample size was 322.

### Study variables

**Dependent variables**

- Cancelations of surgical case.

**Independent variables were;**

- **Socio-demographic variables** (age, sex),
- **Patent related variables** (Patient refusal, Patient absent, lack of investigation and testing, patient unfit for surgery, patient on medication, no need of surgery, medical illness),
- **Staff related variables (**Patient unfit for anesthesia, anesthetist not available, surgeon not available, patient require other surgical work up),
- **Facility related variables (**Shortage of surgical equipment, lack of oxygen, lack of blood, lack of ICU bed, unavailability of anesthesia medication, lack of electricity, and scarcity of equipment) and.
- **Time related variable (**Over scheduled, emergency case priority, previous case prolonged).
- personnel-related factors (surgeon, anesthetist, nurse); incomplete investigation was Independent Variables.

### Operational definition

Elective surgeries scheduled on the final surgeries list for the day or those later added to the list but not done on the day /or were considered cancellations of elective procedures. An operation is referred to as canceled if it is not conducted on the planned date. Elective surgery is defined as non-emergency surgery that is scheduled for a minimum of 24 h in advance [2, 22].

### Data collection tool and procedures

Data were collected by reviewing the daily schedule of elective surgery using a structured questionnaire. A structured questionnaire was developed using patient records and relevant literature to collect demographic, patient, staff, and administrative factors. Reasons for cancellation were determined by interviewing surgical staff (nurses, surgeons, or anesthesiologists) and ward staff on the day of surgery. Data collection was performed by a nurse and supervised by a senior nurse.

### Data quality control

Six qualified professional nurses were chosen based on their education, experience, and ability to communicate in the local language. Two days training was given to data collectors regarding the questionnaire content and how to get data, on interviewing techniques, anthropometric measuring, and data recording from the appropriate bodies.

We ran a pretest on 5% of the entire sample size in the Hadiya Zone of two hospitals two weeks before using it for the final data collection to ensure that the tool was reliable. During the interview and data collection by the data collectors, the investigators kept a close eye on them. Before entering data, each questionnaire was reviewed and checked on a daily basis to ensure that it was comprehensive, accurate, and consistent.

### Data analysis

The data was entered using Epidata V.3 and analyzed using SPSS V.25. To display frequency distributions, descriptive statistics were produced. To find potential variables for multi variable logistic regression analysis, bi variable logistic regression analysis was performed. The multi variable model included all variables that had a *p*-value of less than 0.25 in the bi variable logistic regression analysis. To find the independent variables of the cancellation of surgery procedures, a multi variable logistic regression model was utilized the cases of the cancellation of surgery procedures. The odds ratios (ORs) were calculated using a 95% confidence interval. If the *p*-value was less than 0.05 and the 95% 95% CI did not match the null value, the result was classified as statistically significant. To evaluate the use of multiple logistic models, the Hosmer-Lemeshow goodness of fit assumptions were applied.

### Result

#### Respondents’ sociodemographic characteristics

A total of 313 study participates were scheduled for elective surgical procedures during the study period and giving a response rate of 97.2%. The mean (± SD) age of the study participants was 39.18 (± 10.64) years. Among study participants, 33(39.8%) were within the age group of 30–44 years and 53(64%) were rural residence. The occupation status of 130 (40.4%) of the participants was merchant, and more than half (178, or 55.3%) of the participants were female. More than half of the 255 study participants (69.9%) were able to read and write, and 128 (39.8%) were married. More than half of the respondents’ families (189 or 58.7%) earned more than 2000 ETB (66.66 $) each month **(**Table 1**).**

**Table 1:** Sociodemographic characteristics of participants scheduled for elective surgical procedures in Wolaita Zone, Southern Ethiopia (*N* = 322)

Variables	Category	Frequency of cases schedule	Frequency of canceled cases	Cancellations cases (%)
Sex	Male	148	49	59.1%
	Female	174	34	40.9%
Age groups (years)	< 18	13	11	1.2%
	18–30	90	22	26.5%
	31–43	126	33	39.8%
	44–56	61	21	25.3%
	≥ 57	22	6	7.2%
Occupation	Unemployed	14	11	1.2%
	Employed	93	22	26.5%
	Merchants	122	33	39.8%
	farmers	61	21	25.3%
Place of residences	Yes	206	53	64%
	No	116	30	36%
Alcohol use	Yes	210	53	64%
	No	112	30	36%
Khat chewing	Yes	210	53	64%
	No	116	30	36%
Smoking	Yes	206	53	64%
	No	116	30	36%
Marital status	Married	128	22	26.5%
	Single	93	33	39.8%
	Divorced	61	21	25.3%
	Windowed	22	6	7.2%
Educational status	Primary school	93	22	26.5%
	Secondary school	132	33	39.8%
	Illiterate	57	21	25.3%
	Diploma and above	22	6	7.2%
Annual income (ETB)	< 2000	93	22	26.5%
	2001–4000	130	33	39.8%
	> 4001	59	21	25.3%

### Magnitude of case cancellation and factors associated with it

**Fig. 1:**
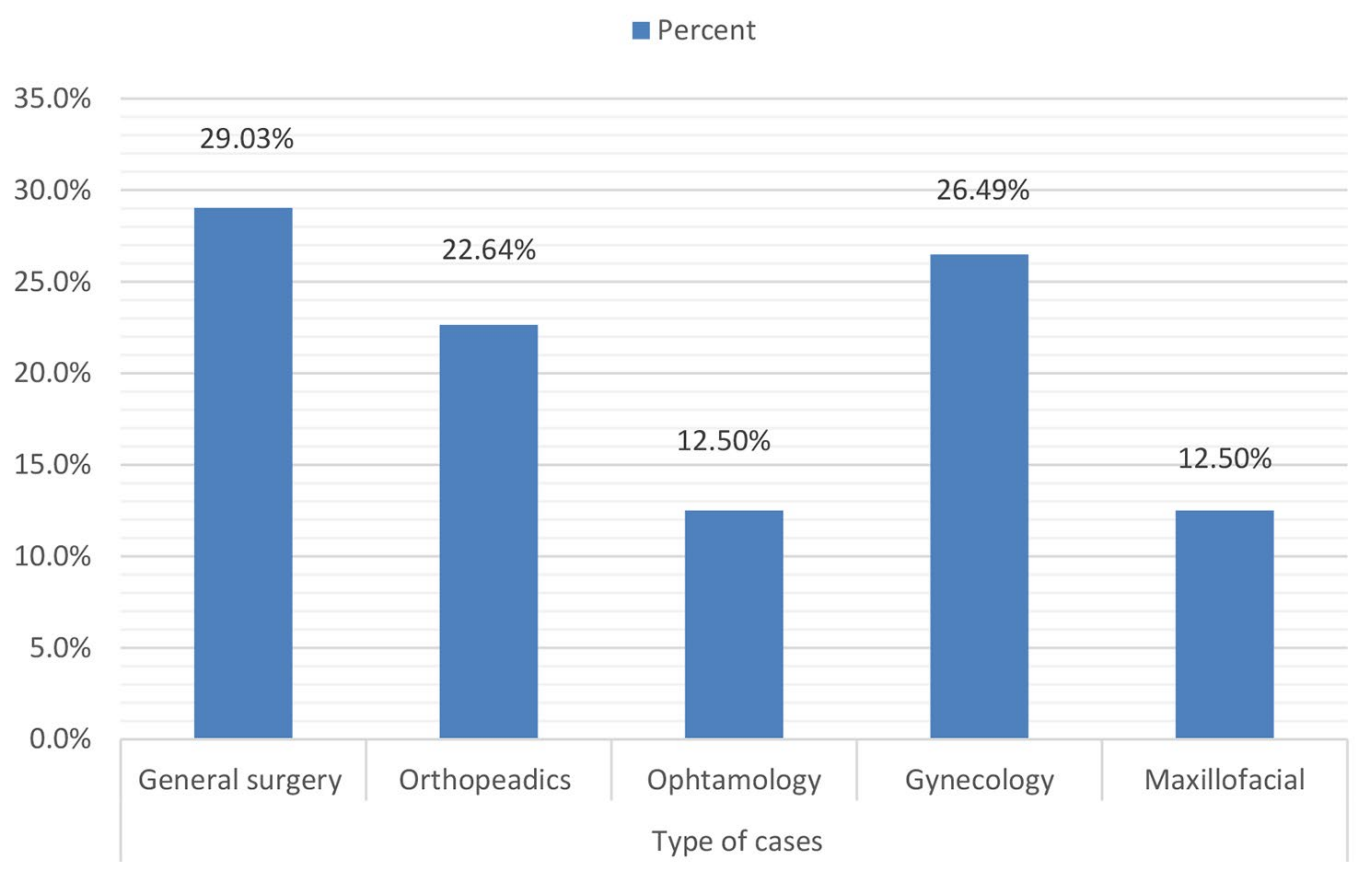
Cancellations of elective surgical procedure among departments of Wolaita Zone hospitals, Ethiopia, 2023

This finding showed that the case cancellation among elective surgical procedures was 22.4% (95% CI: 19.3 -25.9%). Among the total canceled cases, 49(58.3%) were male. Out of the total scheduled elective operation, 250(77.6%) of the patients were operated on their planned day of surgery. According to this finding, the highest number of canceled operations was in the General surgery department (29.03%) followed by Gynecology (26.49%) and orthopedics surgery (22.64%). Ophthalmology and maxillofacial surgery were the least cancellation rate recorded (12.5%) (Fig. 1).

### Reasons to cancel the surgery procedures

The most frequent causes for elective case cancellation were surgeon related factors which accounts 21 cases (29.2%), patient related factors, which accounts 19 cases (26.4%) followed by administrative related factors 17(23.6%) and anesthesia related factor was the least cause of cancellation rate which accounts 16 (19.2%) **(**Table 2**).**

**Table 2:** Reasons of cancellation of elective surgery of participants scheduled for elective surgical procedures in Wolaita Zone, Southern Ethiopia (*N* = 72)

Reason for cancellation	Category	Frequency	Percent (%)
Patient-related	Refusal	4	21.1
	Patient on medication	5	26.3
	Acute and chronic medical illness	3	15.6
	Not fasting for expected hrs.	2	10.2
	Not paid fee	2	10.2
	Absent	3	15.6
Medical illness	A rise in BP/HTN	2	40.0
	Rise in RBS/DM or acute medical illness	3	60.0
Anesthesia related	Unfit for anesthesia	5	55.6
	Abnormal laboratory result	4	44.4
surgeon related	Over-scheduled-elective surgery/case load	7	33.3
	Emergency case priority	6	28.5
	Previous case prolonged	3	14.2
	Surgeon unavailability	2	9.5
	Requires another surgical workup	3	14.2
Administrative related	Shortage of surgical equipment	7	41.2
	Delayed laboratory test	3	17.6
	Lack of oxygen and blood	3	17.6
	Shortage of intensive care unit bed	4	28.2

In bi variable logistic regression analysis, variables such as age, sex, patient refusal, patient absent, lack of investigation and testing, patient unfit for surgery, patient on medication, no need of surgery, place of residences, patient unfit for anesthesia, anesthetist not available, surgeon not available, patient require other surgical work up, shortage of surgical equipment, lack of oxygen, lack of blood, lack of intensive care unit bed, unavailability of anesthesia medication, anesthesia machine and equipment failure, Patient refusal, over scheduled, emergency case priority, and previous case prolonged were significantly linked with cancellation of surgical procedures among elective surgical patients with a *p*-value of < 0.25. In multivariate logistic analysis with a *p*-value of less than 0.05, only five variables (Place of residences, lack of investigation and testing, rise BP or /hypertension, Patient refusal, and age) were significantly associated with the cancellation of surgical procedures among elective surgical cases. There was no correlation between these independent factors according to the VIF.

Study participants from the rural place of residences had 3.48 times the chance of canceling their elective surgical procedures as compare to from urban areas (AOR = 3.48 95% CI: 1.22–9.95). Lack of investigation and testing had 2.33 times more likely to cancel the surgical procedure among elective surgical cases (AOR = 2.33, 95%CI:1.20–4.51) in this study. In the same occasion, study participants in the department of ophthalmology surgery were 2.53 times more likely to have elective surgery cancellation than those in other departments (AOR = 2.53 95% CI:1.52–4.49). Participants with higher blood pressure or rise hypertension were 2.53 times more likely to cancel than their peers to cancel elective surgery (AOR = 2.53, 95% CI:1.52–4.49). On the other hand, Patient refusal is 3.01 times greater chance of having elective surgery cancellation (AOR = 3.01 95% CI:1.22–5.05). Finally, those b/n the ages of 31 and 43 were 1.50 times more likely than others to have hypertension (AOR = 1.50, 95% CI:1.02–2.01) (Table 3**).**

**Table 3:** Bi variable and multi variable logistic regression model of participants scheduled for elective surgical procedures in Wolaita Zone, Southern Ethiopia (*N* = 322)

Variable	Category	Cancellation of surgical procedure		95% CI	
		Yes	No	COR	AOR
Sex	Male	49(33.1%)	99(66.9%)	1	1
	Female	34(19.1%)	140(80.5%)	2.83 (1.17–6.86)	1.31 (0.79–2.15)
Age groups (years)	< 18	11(11.2%)	11(12.6%)	1	1
	18–30	22(26.5%)	22(25.3%)	2.61 (0.91–5.66)	2.18 (0.89–6.97)
	31–43	33(39.8%)	33((37.9%)	**2.99(1.38–6.49)**	2.83(1.17–6.86) *
	44–56	21(25.3%)	21(25.3%)	2.58(1.11–5.56)	1.15(0.39–3.33)
	> 57	6(7.2%)	159(85.9%)	2.99(1.38–6.49)	2.83(0.17–5.86)
Occupation87	Unemployed	11(12.6%)	12(6.5%)	1	1
	Employed	22(25.3%)	14(7.6%)	2.36(1.57–3.27)	1.31(0.79–2.15)
	Merchants	33((37.9%)	16(91.4%)	2.78 (1.11–5.56)	1.31 (0.79–2.15)
	Farmer	21(25.3%)	28(7.0%)	2.26 (0.91–5.66)	2.48 (0.89–6.97)
Annual income (ETB)	< 2000	159(85.9%)	116(63.8%)	1	1
	2001–4000	12(6.5%)	61(15.2%)	2.26(0.91–5.66)	2.48(0.89–6.97)
	> 4001	14(7.6%)	84(20.9%)	2.48(1.11–5.56)	1.15(0.39–3.33)
Patient refusal	Yes	16(91.4%)	272(82.8%)	1	1
	No	16(8.6%)	69(17.2%)	**3.04(1.94–4.78)**	2.33(1.20–4.51) *
Time scheduled	morning	174(94.1%)	336(83.8%)	1	1
	Afternoon	11(5.9%)	65(16.2%)	3.06(1.57–5.95)	0.95(0.39–2.27)
Blood not prepared	Yes	157(84.9%)	260(64.8%)	1	1
	No	28(15.1%)	141(35.2%)	3.12(2.06–4.74)	1.25 (0.72–2.17)
Department of surgical procedures	General surgery	149(80.5%)	235(58.6%)	1	1
	Gynecology	9(4.9%)	19(4.7%)	3.16 (3.16–1.65)	2.21(0.82–5.95)
	Ophthalmology	18(9.7%)	114(28.4%)	**3.73 (3.73–2.05)**	2.53(1.52–4.49) *
	Orthopedics	7(3.8%)	28(7.0%)	3.06 (1.57–5.95)	2.26 (0.91–5.66)
Rise hypertension	No	80(43.2%)	101(25.2%)	1	1
	Yes	105(56.8%)	130(74.8%)	**2.20 (2.20-11.24)**	3.48 (1.22–9.95) *
Marital status	Married	14(10.6%)	14(4.0%)	1	1
	Single	19(14.4%)	43(12.2%)	1.34(0.59–3.04)	0.70(0.25–1.94)
	Divorced	99(75.0%)	196(83.9%)	1.33 (0.13–1.85)	0.95 (0.39–2.27)
	Windowed	14(10.6%)	14(4.0%)	2.54(1.08–5.95)	0.731(0.18–3.03)
Educational status	Primary school	10(5.4%)	61(15.2%)	1	1
	Secondary school	37(20.0%)	77(19.2%)	2.34(1.16–3.74)	1.33 (0.13–1.85)
	Illiterate	118(74.6%)	163(65.6%)	0.31(0.16–0.63)	0.44(0.18–1.07)
	Diploma & above	10(5.4%)	61(15.2%)	3.06 (1.57–5.95)	1.06 (0.57–5.95)
Lack of lab investigation	Yes	104(94.1%)	236(83.8%)	1	1
	No	11(5.9%)	65(16.2%)	**3.54(1.08–5.95)**	**3.01 (1.22–5.05)**
Place of residences	Urban	169(91.4%)	122(82.8%)	1	1
	Rural	16(8.6%)	69(17.2%)	**4.016(2.34–6.87)**	2.87(1.14–7.18) *

Significant at *P < 0.05, 1 = constant, CI = Confidence Interval, COR = Crude odds Ratio, AOR = Adjusted Odds Ratio

## Discussion

This study aimed to assess the magnitude and reasons of elective surgical case cancellation at Wolaita Sodo Zone Hospitals, Southern Ethiopia. This finding showed that the case cancellation among elective surgical procedures was 22.4% (95% CI: 19.3 -25.9%). This study’s findings are in line with studies conducted in Tanzania (21%) [23], Nigeria (20.2%) [24]. This may be approaching of their similarity with sociodemographic factors, sample size, study location, duration, methodological discrepancies, effective hospital management practices, etc.

The finding of this study is higher compared with the study conducted in Ethiopia with meta-analysis 21.4% [25] St. Paul’s Hospital, Addis Ababa (8.9%) [26], Saudi Arabia (7.6%) [27], Zambia 18.8% [28], Sudan (20.2%) [29], America (4.4%) [30], Brazil (6.8%), German (12.7%), Wales (7.6%), New Delhi (17.6%), India (16.49% [31]. This discrepancy may be due to the fact that there are differences in sociodemographic characteristics, sample size, study area, and methodology. In addition, the operating room of this hospital aims to innovate. There is a shortage of operating room equipment and beds, and there is also a shortage of surgeons [23, 32]. It is the largest university-level specialized hospital at the regional level. This increased case flow and referral cases, which in turn reduced operating hours and prioritized urgent case referrals, ultimately leading to higher cancellations.

Our study is lower than studies conducted in Ethiopia (Hawassa (31.6%) [2], Black Lion Hospital (33.9%) [21], Debretabor Hospital (32.1%) [33], Asela Hospital (32.2%) [34], Gondor 33.3% [35], and South Africa (44.5%) [36], India (27.10%) [37], Uganda 28% [38], Oman (26%) [37] and Jimma University teaching hospital (28%). This variation in the magnitude of cancellations might be explained by the fact that: facilities in resource, amount of case overload, surgeons to population ratio, type of hospital and its level, study design, and socioeconomic status of the patient were the possible reasons.

In this study, cancellation rates were highest in general surgery (29.03%), followed by obstetrics and gynecology (26.49%), orthopedics (22.64%), ophthalmology (12.5%), and maxillofacial surgery (12.5%). became. This is supported by studies in Ethiopia and India. The trials at Black Lion Hospital and Orthopedic Surgery in Saudi Arabia had the highest dropout rates across all departments. This study showed that patient and administrative related factors were the two most common cause of cancellation. Cancel surgery due to not fasting (eating) were the major patient related factor and shortage of surgical equipment was the major administrative related factors. The third common cause of cancellation was surgeon related factor, especially over scheduled elective surgery cases were major surgeon related factors. This is supported by conducted studies in Ethiopia, Saudi Arabian, and India.

Study participants from the rural place of residences had 3.48 times the chance of canceling their elective surgical procedures as compare to from urban areas.

One possible cause is the lack of transportation. Again, the majority of Ethiopians live in rural areas and have few resources. People in rural areas face major obstacles due to the enormous distances between their communities and the nearest health facilities. Depending on the region, traffic and lack of road infrastructure can be a problem. Transportation costs can also influence the choice of surgical technique.

Lack of investigation and testing had 2.33 times more likely to cancel the surgical procedure among elective surgical cases in this study. In the same occasion, study participants in the department of ophthalmology surgery were 2.53 times more likely to have elective surgery cancellation than those in other departments. This May be due to lack of benefits for seniors.

Participants with higher blood pressure or rise hypertension were 2.53 times more likely to cancel than their peers to cancel elective surgery. This is consistent with findings from a study conducted in Singapore [30]. A possible explanation for this is that hypertension increases cardiovascular and cerebrovascular events, hemorrhage and mortality in the preoperative and postoperative period and is treated before major elective noncardiac or cardiac surgery. that there is a need. Because of this, the medical professional or medical team canceled the surgery to prevent further complications. If the systolic blood pressure is 180 mmHg or higher or the diastolic blood pressure is 110 mmHg or higher, it is recommended to postpone the planned surgery due to the risk of complications.

Elective noncardiac surgery and cardiac surgery. Because of this, the medical professional or medical team canceled the surgery to prevent further complications. If the systolic blood pressure is 180 mmHg or higher or the diastolic blood pressure is 110 mmHg or higher, it is recommended to postpone the planned surgery due to the risk of complications [30, 39].

On the other hand, Patient refusal is 3.01 times greater chance of having elective surgery cancellation. Finally, those b/n the ages of 31 and 43 were 1.50 times more likely than others to have hypertension. This difference may be due to these age groups. Patients aged 0 to 10 years and older are more likely to be preoperatively assessed and prioritized prior to the day of surgery.

### Strengths and limitations

Both primary and secondary data were used to obtain complete information about study participants. This study will further evaluate factors that have not been previously evaluated.

However, this study has certain limitations. Restrictions related to cross-sectional studies may apply. The study included only those scheduled for elective surgery and did not include mothers who delivered children by elective caesarean section. This study was also limited to public hospitals and excluded private hospitals, thus underestimating the results of the study.

## Conclusion

In this study scheduled of elective surgery cancellation was high. The contributing factors to the cancellation of elective surgery were place of residences, Lack of lab result, ophthalmology dept, HTN, Patient refusal, and age.

This finding shows that, elective surgeries were frequently canceled on the scheduled date of surgery. It is well known that most reasons for cancelling are avoidable and can be prevented in various ways. This can help to reduce wasting of resources and valuable time to provide more healthcare services to the public if patients with medical concerns are identified early and referred for an aesthetic evaluation immediately after a planned surgery. All hospital administrators and other stakeholders should ensure proper planning, adequate information for scheduled patients, provision of necessary operating room equipment, including blood tests, and clear communication with operating room teams was strongly recommended. Improve operating room efficiency and provide appropriate patient counseling on schedule. Evidence-based and timely reporting by monitoring teams was therefore recommended to reduce the number of cancellations.

## Data Availability

The datasets used and/or analyzed during the current study available from the corresponding author without undue reservation.

## Abbreviations

OT: Operation Theater
WSUCSH: Wolaita Sodo University Compressive Specialized Hospital
